# Challenges of the Maternal Role in Women with Schizotypal Personality Disorder

**DOI:** 10.1192/j.eurpsy.2025.1912

**Published:** 2025-08-26

**Authors:** N. Curk Fišer

**Affiliations:** 1 Intensive Care Unit, University Psychiatric Clinic Ljubljana, Ljubljana, Slovenia

## Abstract

**Introduction:**

Children born to mothers with mental health disorders are at a significantly increased risk of developing insecure attachment patterns.

**Objectives:**

Demonstrate with clinical case that mothers with schizotypal personality disorder tend to be less sensitive and responsive, and more intrusive in their interactions and caregiving (Høivik et al. BMC Psychiatry Int 2018; 18, 198; Willinger et al. ANZJP Int 2002, 36, 5; Vebeke et al. al. APA Int 2017, 8 54-63; Ripoll et al.).

**Methods:**

The clinical case presented below highlights functional decline in a woman with schizotypal personality disorder during pregnancy and the transition to motherhood.

**Results:**

The 39-year-old mother of a 16-month-old son, employed as a saleswoman, has been in outpatient psychiatric treatment for 6 years. She has been hospitalized 6 times, 4 of which occurred in the year after childbirth.

Her psychiatric history began at age 33, when she was first admitted due to an impulsive outburst, involving aggression toward objects. Psychological evaluation revealed impulsivity alongside paranoid ideation. Treatment (aripiprazole, quetiapine, and paroxetine) led to clinical improvement, and she continued psychotherapy for psychosis. For 5 years, she maintained regular employment, entered a relationship, and became pregnant, at which point she discontinued pharmacotherapy.

At 32 weeks of pregnancy, she became disorganised, refusing food and gynecological examinations, leading to psychiatric hospitalization. A combination of haloperidol, quetiapine, and lorazepam stabilized her condition, although negative symptoms, cognitive impairments and schizotypal features persisted. She gave birth to a healthy son via planned cesarean section and continued postnatal care under a psychiatrist and clinical psychologist.

Within the year postpartum, she experienced 4 additional hospitalizations, primarily due to mood destabilization linked to non-adherence to oral and depot pharmacotherapy. Each time, her condition improved upon reintroduction of therapy. By her penultimate discharge, a social coordinator was appointed, as she struggled with childcare, expressing emotional detachment from her child. Her fourth hospitalization was characterized by disorganization and impulsivity, after which she ended her relationship with her partner. Currently, she resides with her mother and remains on quetiapine, clozapine, and aripiprazole. With her consent, her partner and his mother assumed childcare responsibilities. The patient reports feeling emotionally relieved and stable, and has agreed to resume depot pharmacotherapy.

**Conclusions:**

This case underscores the importance of early identification and timely intervention in perinatal psychiatry. Effective treatment of maternal mental illness, doesn’t necessarily lead to secure attachment formation between mother and child, nor does it guarantee better cognitive or socio-emotional outcomes in the offspring.

**Disclosure of Interest:**

None Declared

